# When Time Is Not on Your Side: Delayed Recognition of Hand Compartment Syndrome After a Fracture

**DOI:** 10.1155/cro/1830898

**Published:** 2025-04-15

**Authors:** Edmund M. Bediako, Geraldine K. Mould, Eadbert Nortey, Abigail Mills-Annoh, Priscilla Kyei-Baffour, Ama Ekem, Jemima C. A. Clarke, Eugene F. E. K. Apaloo, Susan Quartey-Papafio, Kwame Ekremet

**Affiliations:** Emergency Department, University of Ghana Medical Centre (UGMC), Accra, Ghana

**Keywords:** compartment syndrome, forearm injury, fracture, gangrene

## Abstract

Compartment syndrome of the forearm is a well-described clinical entity, but only a few case reports on hand compartment syndromes have been reported due to its rarity. The early recognition and treatment of this potential threat to the limb are important in order to prevent permanent disability and other life-threatening complications. This is a documented case report on the late presentation of compartment syndrome of the hand following a fall.

## 1. Introduction

Compartment syndrome is a potentially limb-threatening condition characterized by elevated intercompartmental pressures, resulting in impaired local vascular circulation and tissue function [1]. We present a case of a 54-year-old woman diagnosed with compartment syndrome of the hand following a left distal radial fracture with radiocarpal joint dislocation. This diagnosis was made based on clinical findings identified at the Emergency Department (ED) of the University of Ghana Medical Centre (UGMC). Given the severity of her condition and the irreversible tissue damage, a below-elbow amputation of the left forearm was promptly performed. This case highlights the potential complications of fractures and emphasizes the importance of prompt recognition and timely medical intervention. Through this report, we aim to contribute to the existing literature on the diagnosis and management of compartment syndrome of the hand, with a focus on the unique clinical challenges encountered in a resource-limited setting.

## 2. Case Presentation

A 54-year-old woman sought medical attention at the ED of the UGMC with a painful and swollen left hand of a week's duration. She is known to be living with hypertension and bipolar disorder and is compliant on her medications. She was well until a week prior to presenting when she slipped and fell on her left hand. Initially seeking care from a traditional medicine practitioner, who applied daily topical herbal preparations for a week, she subsequently visited ED with a relative who observed significant swelling and discoloration in her left hand.

**Figure 1:**
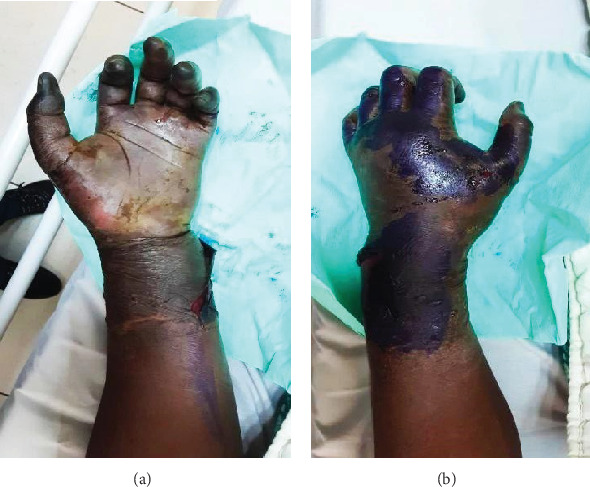
(a) Palmar and (b) dorsal aspects of the patient's left hand at presentation demonstrate a mottled appearance of all five digits.

Upon physical examination, her left hand exhibited marked swelling, and the fingertips appeared cold and dark (gangrenous) with multiple blisters at the wrist area, extending proximally about 5–7 cm. Sensation was intact in the thenar and hypothenar regions but absent in the fingertips. Radial pulses were present bilaterally, and a clear demarcation area about 3 cm above the wrist (Figure 1) was observed. Her vital signs at presentation were as follows: temperature of 36.6°C, blood pressure of 152/86 mmHg, heart rate of 83 beats per minute, and respiratory rate of 18 cycles per minute with 99% oxygen saturation on room air.

**Figure 2:**
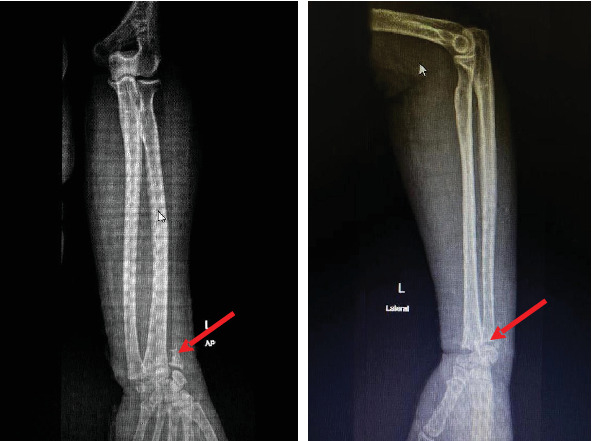
Plain X-ray of the left hand (AP and lateral) showing a left distal radial fracture with radiocarpal joint dislocation.

Laboratory results at the time of admission were as follows: Hb 9.1 g/dL, WBC 8.23 × 109/L, Plt 292 × 109/L, with normal kidney function. A plain X-ray of the left hand revealed a left distal radial fracture with radiocarpal joint dislocation (Figure 2). A diagnosis of left-hand gangrene secondary to compartment syndrome due to a fracture following a fall was made. The orthopedic team counseled the patient for a below-elbow amputation of the left forearm, which was performed the next day after admission to the ward. The patient made a full recovery and was discharged 2 days later.

## 3. Clinical Discussion

Compartment syndrome results from elevated intercompartmental pressures, impairing vascular circulation and muscle and nerve function. While commonly observed in lower limbs and forearms, hand involvement is rare. The hand is made up of 11 compartments: four dorsal interossei, three volar interossei, thenar, hypothenar, adductor, and midpalm compartments. It is supplied by branches from the radial and ulnar arteries, respectively [1].

Compartment syndrome occurs when tissue or interstitial pressure within the compartment exceeds venous capillary pressure, leading to a narrowed arteriovenous perfusion gradient and microvascular collapse. This results in decreased muscle perfusion, ischemia, and subsequent local hypoxia, nerve dysfunction, and muscle necrosis [2]. In compartment syndrome, muscle damage precedes nerve impairment and is reversible for the first 4 h. The onset of muscle necrosis duration is uncertain, with studies suggesting between 8 and 12 h [3]. Happenstall et al. demonstrated that interstitial pressures exceeding 40 mmHg for at least 8 h resulted in permanent tissue necrosis and nerve conduction arrest [4]. Causes of increased intracompartmental pressure include edema, hematoma formation, and fracture, while external compression factors may include circumferential burns, prolonged periods of immobilization, and tight wrapping [3].

Compartment syndrome is usually a clinical diagnosis, especially in low-resource countries with limited access to specialized medical equipment. In uncertain cases, intracompartmental pressure measurement is a useful supplemental tool [5]. Pain is the most common symptom, exacerbated when involved muscles are passively moved [6]. The pain also grows progressively worse and out of proportion in severity when compared to the injury. Other symptoms may include swelling over the compartment, pulselessness, and pallor, which often suggest there is arterial involvement through either arterial compression or transection. Paresthesias and paralysis are usually late signs, indicating nerve ischemia.

Comparing compartment syndromes at other sites to the hand, hand compartment syndrome generally lacks clinically significant findings such as sensory deficits, as the nerves innervating the hand are not located within the hand compartments [1]. This may explain why this patient retained sensation despite presenting late to the hospital. Direct measurement of intracompartmental pressure is particularly useful in certain circumstances, such as in intoxicated, obtunded, uncooperative, or mentally altered patients. When intracompartmental pressures exceed 30 mmHg or are within 30 mmHg of diastolic blood pressure, urgent evaluation for possible fasciotomy and limb salvage is necessary.

Early recognition of compartment syndrome allows for timely fasciotomy, which can prevent irreversible tissue damage and potentially reduce amputation rates [7]. However, failure to promptly diagnose and treat compartment syndrome may lead to nonviable limbs and subsequent amputation [8].

## 4. Conclusion

Hand compartment syndrome, with various etiologies, is a relatively uncommon condition. Urgent diagnosis and management are essential to prevent significant functional morbidity and disability. Recognizing it early is crucial for initiating treatment and minimizing functional loss in the affected limb. This case underscores the critical importance of timely medical intervention. Failure to promptly diagnose and initiate treatment, followed by adequate postoperative rehabilitation, often results in adverse outcomes, including necrosis of muscles and other tissues, ischemic contraction of muscles (Finochietto contraction), nerve paralysis, and amputation, as seen in this patient. The successful outcome of a below-elbow amputation, recommended and promptly performed by the orthopedic team, led to the patient's full recovery.
